# Association between Hepatic Venous Congestion and Adverse Outcomes after Cardiac Surgery †

**DOI:** 10.3390/diagnostics12123175

**Published:** 2022-12-15

**Authors:** Csaba Eke, András Szabó, Ádám Nagy, Balázs Szécsi, Rita Szentgróti, András Dénes, Miklós D. Kertai, Levente Fazekas, Attila Kovács, Bálint Lakatos, István Hartyánszky, Kálmán Benke, Béla Merkely, Andrea Székely

**Affiliations:** 1 Rácz Károly School of PhD Studies, Semmelweis University, Ulloi Ut 26, 1085 Budapest, Hungary; 2 Faculty of Medicine, Semmelweis University, Ulloi Ut 26, 1085 Budapest, Hungary; 3 Department of Anesthesiology, Vanderbilt University Medical Center, Nashville, TN 37212, USA; 4 Heart and Vascular Center, Semmelweis University, Varosmajor Utca 68, 1123 Budapest, Hungary; 5 Faculty of Health Sciences, Semmelweis University, Vas Utca 17, 1088 Budapest, Hungary

**Keywords:** Doppler ultrasound, hepatic venous flow, fluid balance, cardiac surgery

## Abstract

Introduction: Hepatic venous flow patterns reflect pressure changes in the right ventricle and are also markers of systemic venous congestion. Fluid management is crucial in patients undergoing cardiac surgery. Methods: Our goal was to determine which factors are associated with the increased congestion of the liver as measured by Doppler ultrasound in patients undergoing cardiac surgery. This prospective, observational study included 41 patients without preexisting liver disease who underwent cardiac surgery between 1 January 2021 and 30 September 2021 at a tertiary heart center. In addition to routine echocardiographic examination, we recorded the maximal velocity and velocity time integral (VTI) of the standard four waves seen in the common hepatic vein (flow profile) using Doppler ultrasound preoperatively and at the 20–24th hour of the postoperative period. The ratios of the retrograde and anterograde hepatic venous waves were calculated, and the waveforms were compared to the baseline value and expressed as a delta ratio. Demographic data, pre- and postoperative echocardiographic parameters, intraoperative variables (procedure, cardiopulmonary bypass time), postoperative factors (fluid balance, vasoactive medication requirement, ventilation time and parameters) and perioperative laboratory parameters (liver and kidney function tests, albumin) were used in the analysis. Results: Of the 41 patients, 20 (48.7%) were males, and the median age of the patients was 65.9 years (IQR: 59.8–69.9 years). Retrograde VTI growth showed a correlation with positive fluid balance (0.89 (95% CI 0.785–0.995) c-index. After comparing the postoperative echocardiographic parameters of the two subgroups, right ventricular and atrial diameters were significantly greater in the “retrograde VTI growth” group. The ejection fraction and decrement in ejection fraction to preoperative parameters were significantly different between the two groups. (*p* = 0.001 and 0.003). Ventilation times were longer in the retrograde VTI group. The postoperative vs. baseline delta VTI ratio of the hepatic vein correlated with positive fluid balance, maximum central venous pressure, and ejection fraction. (B = −0.099, 95% CI = −0.022–0.002, *p* = 0.022, B = 0.011, 95% CI = 0.001–0.021, *p* = 0.022, B = 0.091, 95% CI = 0.052–0.213, *p* = 0.002, respectively.) Conclusion: The increase of the retrograde hepatic flow during the first 24 h following cardiac surgery was associated with positive fluid balance and the decrease of the right ventricular function. Measurement of venous congestion or venous abdominal insufficiency seems to be a useful tool in guiding fluid therapy and hemodynamic management.

## 1. Introduction

Objective assessment of hemodynamic conditions is fundamental to guide the clinical management of the cardiac surgery patient during the postoperative period [1,2,3]. Meticulous fluid management is crucial in patients undergoing cardiac surgery, particularly in those with heart failure; prolonged operative and aortic cross-clamp time; or preexisting kidney, lung, or liver dysfunction [4,5]. Recent studies suggested that abdominal congestion can lead to or worsen renal and hepatic dysfunction and thus increase the risk of postoperative complications and lead to higher resource utilization [6,7]. Postoperative fluid overload is associated with prolonged mechanical ventilation, higher vasoactive, inotropic support, and mortality [6,7,8]. The changes in the venous flow patterns of different organs due to congestion can be measured and quantified by several ultrasound techniques [9,10,11].

Many physicians performing point of care ultrasound assessment (POCUS) usually use inferior vena cava (IVC) measurements to predict fluid status. However, there are more venous structures that clinicians could evaluate. Using solely IVC measurements can lead to inaccurate results because they do not accurately represent the patient’s preload conditions of the left ventricle. The IVC can also be dilated in conditions such as tricuspid or mitral insufficiency, pulmonary hypertension, or in athletes. Many recent protocols, such as the venous excess ultrasound examination (VExUS) use several hepatic, portal, and renal structures for Doppler ultrasound analysis [12,13]. The renal venous Doppler pattern is seen as a continuous monophasic flow. As venous congestion increases, there is a decrease of the systolic component of the wave with progression to a biphasic pattern and later a complete absence of systolic flow can be seen [14]. The portal venous flow is normally monophasic with little to no variation. As venous congestion increases, increasing amounts of pulsatility can be detected in the flow pattern. The hepatic venous flow is composed of a systolic (S), a diastolic (D) and two retrograde waves (A and V). As venous congestion increases, a decrement of the S wave (smaller than D) and an increment of the retrograde waves (A and V) can be seen [15,16].

However, the influence of postoperative interventions, such as fluid therapy and the use of vasoactive drugs, or the probably significant effect of positive pressure ventilation, has not been analyzed in relation to venous return.

The aim of our study was to investigate the postoperative factors that might be associated with increased hepatic venous congestion during the postoperative period in patients undergoing cardiac surgery. The investigation focused on the changes and relationships among hepatic waveforms and echocardiographic parameters, ventilator settings, fluid and vasoactive medications, and laboratory parameters of renal and liver functions during the first 24 h. Our goal in our prospective observational study was to determine which factors are associated with the increased congestion of the liver as measured by Doppler ultrasound in patients undergoing cardiac surgery.

## 2. Methods

### 2.1. Study Design

**Figure 1:**
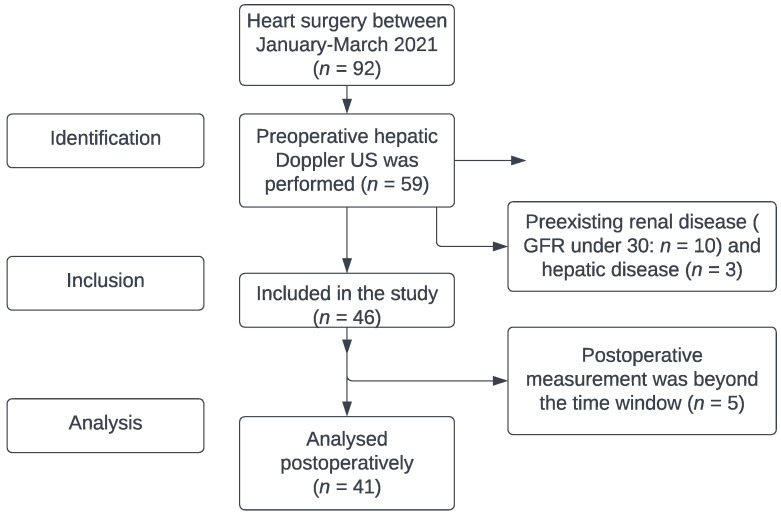
Flowchart by STROBE statement (identification–inclusion–analysis). Detailed information in Supplementary Materials File S1.

The study results are reported according to the STROBE statement. The filled form can be found in Supplementary Materials File S1. Our study received approval from the Institutional Review Board of Semmelweis University (IRB 141/2018), and it was registered on ClinicalTrials.gov (NCT02893657). Each patient who agreed to participate signed an informed consent form before the first investigation. In this prospective, observational study, 41 patients undergoing cardiac surgery between January 2021 and March 2021 were enrolled. Exclusion criteria were preoperative chronic kidney disease (defined as GFR under 30 mL/min/1.73 m^2^), hepatic cirrhosis, and portal vein thrombosis. (Figure 1)

### 2.2. Definitions and Measurements (Variables and Data Sources and Grouping)

Demographic data, perioperative echocardiographic parameters, and intraoperative variables (procedure type, cardiopulmonary bypass time) were collected. Postoperative factors (fluid balance, vasoactive requirement, ventilation time and parameters—respiratory rate, tidal volume, positive end-expiratory pressure, fraction of inhaled oxygen, and perioperative laboratory parameters (liver, kidney function, albumin) were also used in the analysis. Laboratory results were collected during the preoperative period and on the first, second, and third postoperative days. In addition, information on the predictors of the European System for Cardiac Operative Risk Evaluation II score (EuroSCORE II) was used and [17] the Vasoactive Inotrope score (VIS) was calculated [18]. EuroSCORE II is a widely used risk stratification system for the cardiac surgical population using patient (clinical preoperative state, mobility), operation (urgency, operation at thoracic aorta), and cardiac risk factors (LV function, recent myocardial infarction, pulmonary hypertension). VIS is calculated using vasopressor (norepinephrine, epinephrine, vasopressin) and inotropic (dobutamine, dopamine, levosimendan, milrinone) medication doses.

### 2.3. Ultrasound Analysis

The ultrasound examinations were performed by board-certified cardiologists (AK, BL) and were recorded on the same machine and analyzed by the same person after completion of the study (CE). Standard 2D parameters recorded were ejection fraction, tricuspid annular plane systolic excursion, atrial and ventricular diameters, and the occurrence of any valvular pathology. The physicians managing the patients during the postoperative period were blinded to the results of the study-specific measurements.

**Figure 2:**
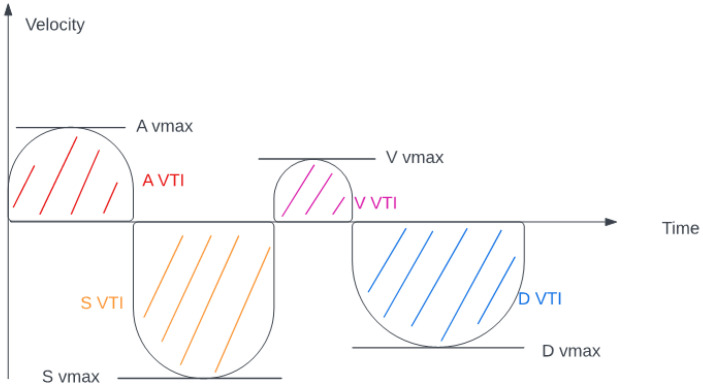
Hepatic venous flow (velocity-time integral and maximal velocity explanation).

**Figure 3:**
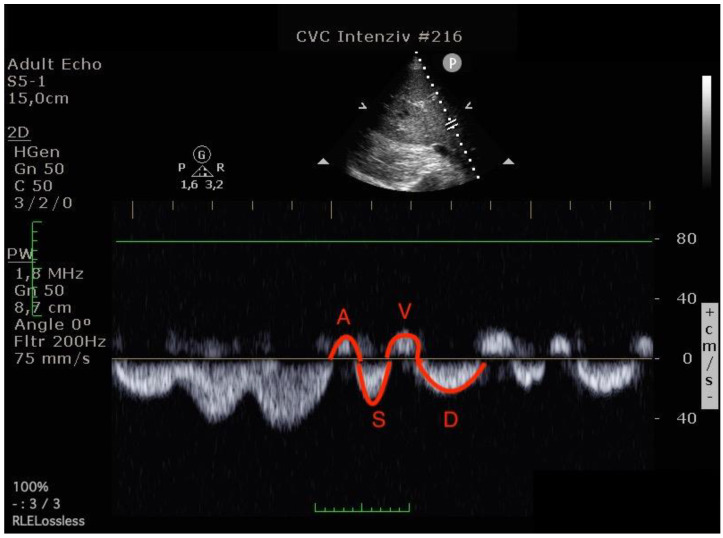
Hepatic venous flow pattern (The left image was done before operation, the right image in the postoperative 24th hour. On the postoperative image, A and V waves appear increased, while the S wave is smaller than the D wave. (A VTI: 6.47 to 8.29, S VTI: 12.31 to 8.12, V VTI: 5.98 to 7.98 and D VTI: 11.12 to 9.39).

**Figure 4:**
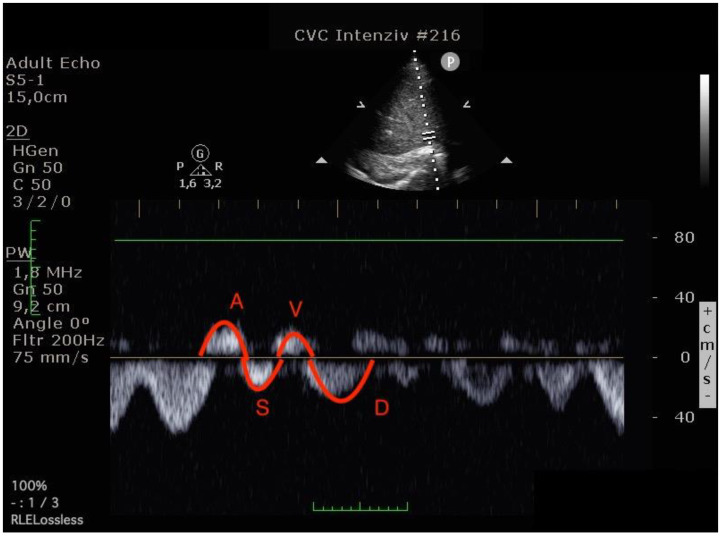
Hepatic venous flow pattern (The left image was done before operation, the right image in the postoperative 24th hour. On the postoperative image, A and V waves appear increased, while the S wave is smaller than the D wave. (A VTI: 6.47 to 8.29, S VTI: 12.31 to 8.12, V VTI: 5.98 to 7.98 and D VTI: 11.12 to 9.39).

Blood flow was measured in the common hepatic vein right before draining into the inferior vena cava using pulse-waved Doppler ultrasound. The normal hepatic vein waveform has four components: a retrograde A, an anterograde S, a transitional V (which may be anterograde, retrograde, or neutral), and an anterograde D wave [15,19]. (Figure 2) We recorded the maximal velocities and velocity-time integrals (VTI) of the standard four waves (A, S, V, D) [19,20] (Figure 3 and Figure 4). The baseline ratios of the retrograde and anterograde waves were calculated preoperatively and their change in the postoperative measurement (20–24 h after surgery), is expressed as a delta ratio. The ratios of retrograde to anterograde VTIs were also calculated.

### 2.4. Outcome Variables

We investigated the correlations between echocardiographic parameters and the changes in hepatic venous flow parameters. Changes of serum creatinine, bilirubin, and transaminase levels from their baseline values were also correlated with changes of the VTI ratios. Fluid overload and the amount of vasoactive support were analyzed both as continuous parameters and comparison between groups who had growth in the VTI ratio compared to those without growth.

### 2.5. Statistical Analysis

Normality was assessed using the Kolmogorov–Smirnov test. Normally distributed values were described as means and standard deviations (SD) and skewed distributions as medians and interquartile ranges (interquartile range 25–75) and were compared using the Mann–Whitney U test. Continuous variables were first expanded with restricted cubic splines and were only used in linear form if the deviation from linearity was not significant, as indicated by the global F test (*p* > 0.05). Multivariable models were tested for multicollinearity. To test the diagnostic ability of the binary (hepatic venous congestion and non-VTI growth) classifier system, receiver operating characteristic curves were generated. Statistical tests were two-sided, and *p* < 0.05 was considered statistically significant. Statistical analyses were performed with SPSS software, Version 27.0 (IBM, Armonk, NY, USA).

## 3. Results

**Figure 5:**
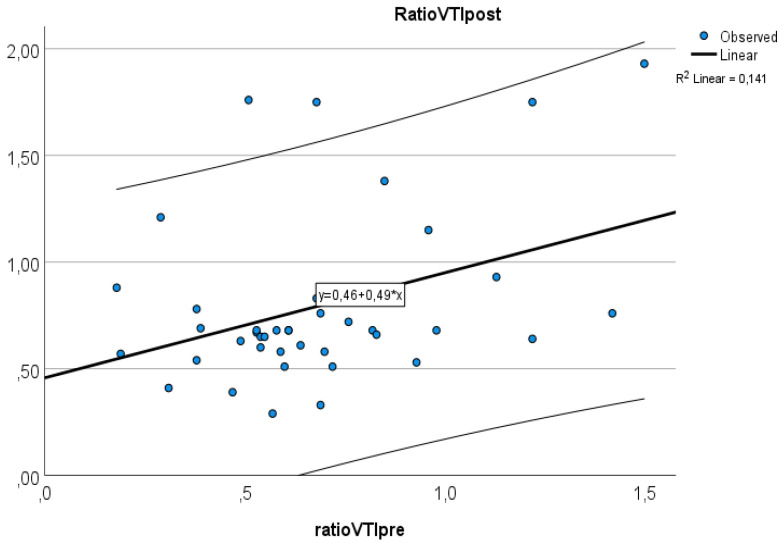
Relationship between preoperative and postoperative VTI ratios.

**Figure 6:**
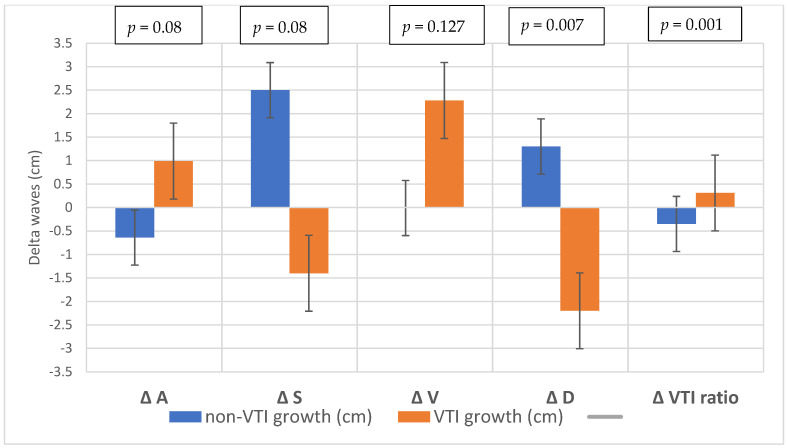
Changes in the waves during the preoperative–postoperative period.

**Figure 7:**
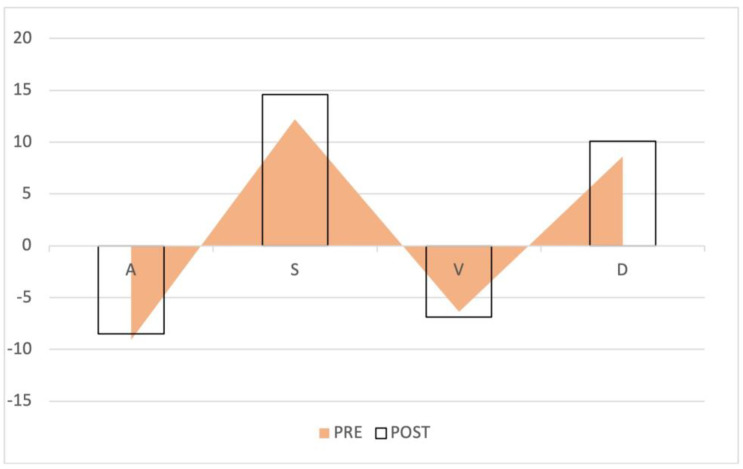
Waveform changes in the no retrograde VTI growth group. The triangles represent the preoperative waves, the columns the postoperative waves. The anterograde S and D waves increased, while the retrograde waves remained the same.

**Figure 8:**
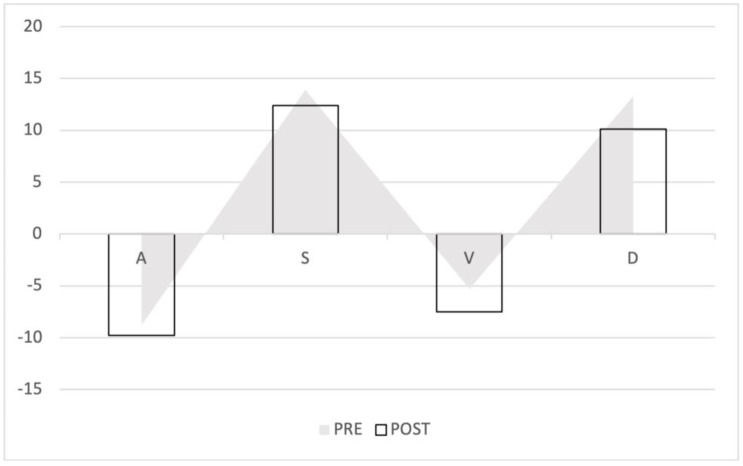
Waveform changes in the retrograde VTI growth group. The triangles represent the preoperative waves, the columns the postoperative waves. The anterograde waves (S and D) decreased, the retrograde flow increased (A and V).

Of the 41 patients, 20 (48.7%) were male. The median age of the patients was 65.9 years (IQR: 59.8–69.9 years). The baseline demographic and clinical characteristics of the study population are shown in Table 1. The average increase in the retrograde/anterograde wave VTI ratio was 0.04 (from 15.5/20.8 = 0.77 to 19.7/24.4 = 0.81), which means that the proportion of retrograde VTIs increased by 4% on average in the first 24 postoperative hours. After examining the waves, we found that the majority of the population had an increased retrograde VTI growth (24/41 = 59%), as indicated by an increase in the VTIs of the retrograde waves compared to the baseline values. After analyzing the retrograde/anterograde VTI ratios, the preoperative value showed a significant correlation with the postoperative VTI ratio (Figure 5). R square was 0.147 F 6.21 *p* = 0.017. In the retrograde VTI growth group, delta D decreased, and there was a tendency toward an increase in delta A and delta V waves (Figure 6, Figure 7 and Figure 8).

After comparing the echocardiographic parameters between the two subgroups, the postoperative right ventricular and atrial diameters were significantly greater in the “retrograde VTI growth” group (Table 2). The ejection fractions and the decreases in ejection fractions were significantly different between the two groups. (*p* = 0.001 and 0.009).

We analyzed the vasoactive and inotropic scores, respiratory parameters, central venous pressures (CVP), and fluid balance.

Ten patients needed inotropic support on the first postoperative day. Among the standard laboratory parameters, postoperative GFR values were lower, and blood urea nitrogen and bilirubin levels were higher in the “VTI ratio increased” subgroup. (Supplementary Materials File S3).

After analyzing the respiratory parameters and ventilation times, we found that the VTI growth subgroup had longer ventilation duration (the median value was over 24 h) and needed higher positive end-expiratory pressures (*p* = 0.003) (Table 3).

Retrograde VTI growth showed a 0.89 (95% CI 0.785–0.995) C-index relationship with positive fluid balance.

In the univariable linear regression model, the postoperative/baseline delta VTI ratio of the hepatic vein correlated with fluid balance, maximum central venous pressure, and delta ejection fraction (Table 4). There was no correlation between the bilirubin and creatinine levels and VIS scores. In the multivariable linear regression model, none of the variables had independent association with the delta VTI ratio.

## 4. Discussion

We found that higher preoperative retrograde VTI ratios were associated with higher postoperative retrograde VTI ratios. In the first 24 h, the increase of the retrograde flow in the hepatic veins was associated with worse ejection fraction, higher positive fluid balance, increased right atrial and ventricular diameters, and higher central venous maximum filling pressures.

The link between congestive heart failure, right ventricular failure, and liver dysfunction has been highlighted in the past decades [21,22,23]. Right ventricular failure can lead to hepatic dysfunction, ranging from mild enzyme elevations to severe hepatic fibrosis [7,24,25]. Congestion can impair kidney function and can cause gastrointestinal ischemia due to high backward pressures and lower effective organ pressure gradients. The change in the renal venous flow pattern (but not the right atrial pressure) was associated with a higher one-year mortality in patients with advanced heart failure [26]. In our study, we also found a close relationship between the presence of preexisting retrograde flow and an increased retrograde flow after cardiac surgery. The increase in the retrograde flow was associated with the severity of fluid overload, reduced ejection fraction, and reduced right-sided cardiac function. It is not clear whether fluid overload leads to an increase in retrograde flow, or it is caused by preexisting venous insufficiency (such as varicosities in the lower limb) or both [26].

Measuring venous flow patterns with ultrasound is an easy and noninvasive method that can help guide fluid management. These new diagnostic concepts, such as the inspection of the venous flow patterns in the renal and hepatic veins, can help in the early detection of fluid overload. It can be different from and nonparallel with right-sided cardiac dysfunction, and it cannot be predicted by the volume or cardiac output changes on the arterial side [14]. In our study, we measured the VTIs instead of the maximum velocities of the waveforms, which might be more useful for volume estimation. Using ratios for the expression of the retrograde and anterograde flows has the advantage of negating the effects of vessel shape and diameter and might reduce potential individual errors.

Fast track management after cardiac surgery can be beneficial, as early return of spontaneous breathing and extubation will promote negative thoracic pressure, increasing the venous return, preloading, and helping to maintain cardiac output [27]. Prolonged positive pressure ventilation is associated with a reduction and redistribution of cardiac output, decreased splanchnic blood flow, and reduced blood supply to the liver [27,28,29]. Our data also support that the level of PEEP might be associated with a decrease in the anterograde flow in the hepatic veins.

In the postoperative period, preload optimization can lead to fluid overload, particularly in low cardiac output states, preexisting or newly developed right-sided heart failure, and pulmonary hypertension. In other words, monitoring the hepatic venous waveform can help in the early detection of abdominal congestion states and fluid overload.

## 5. Conclusions

Measurement of venous congestion or venous abdominal insufficiency seems to be an important tool in guiding fluid management and vasoactive therapy [29]. Volume and pressure monitoring on the arterial side does not yield sufficient hemodynamic data, and right atrial pressure monitoring alone provides inadequate information about the venous side of the circulation. Monitoring abdominal venous waveforms is an easy and inexpensive noninvasive method that can help to detect fluid overload earlier.

## Figures and Tables

**Table 1:** Demographic and clinical parameters (operation time and risk factors).

Parameters	All Patients (*n* = 41)	No Retrograde VTI Growth (*n* = 17)	Retrograde VTI Growth (*n* = 24)	*p*
Age (years)	65.9 (10.8)	63.7 (11.3)	67.1 (10.4)	0.272
Weight (kg)	72.0 (10.2)	73.4 (11.1)	71.1 (10.3)	0.845
Diabetes	19 (46.3%)	9 (53%)	10 (41.6%)	0.53
Sex female	21 (51.2%)	9 (53%)	12 (50%)	0.466
EUROSCORE II	4.7 (1.3)	4.9 (0.9)	4.5 (1.0)	0.197
NYHA III/IV	24 (58.5%)	10 (58.8%)	14 (58.3%)	0.456
Operation time (min)	182.4 (39.1)	178.1 (41.1)	188. 8 (39.1)	0.88
Aorta cross-clamp time (min)	47.8 (7.1)	40.8 (9.1)	48.1 (7.6)	0.73
Operation type				
MVR	9 (22%)	3 (17.6%)	6 (25%)	0.234
AVR	14 (34.1%)	6 (35.2%)	8 (33.3%)	0.199
CABG	15 (36.6%)	7 (41.1%)	8 (33.3%)	0.342
Combined	3 (7.3%)	1 (5.9%)	2 (8.4%)	0.544

NYHA: New York Heart Association; MVR: mitral valve repair; AVR: aortic valve repair; CABG: coronary artery bypass graft; EUROSCORE II: European System for Cardiac Operative Risk Evaluation II.

**Table 2:** Preoperative and postoperative echocardiographic parameters (mean or median (IQR or SD)).

Echo parameter	Non-VTI Growth (*n* = 17)	VTI Growth (*n* = 24)	*p*	Non-VTI Growth (*n* = 17)	VTI Growth (*n* = 24)	*p*
	Preoperative			Postoperative		
EF	53.7 (10.5)	51.5 (12.4)	0.277	59.4 (11.2)	48.5 (10.7)	0.001
TAPSE (mm)	24.6 (5.1)	22.6 (5.9)	0.127	14.3 (4.2)	15.2 (5.4)	0.251
LVEDD (mm)	51.1 (9.2)	53.1 (10.4)	0.255	44.6 (4.1)	47.4 (6.8)	0.08
LVESD (mm)	39.9 (14.1)	38 (10.1)	0.431	28.4 (9.1)	33.3 (8.7)	0.022
RV (mm)	32.7 (4.4)	32.9 (3.9)	0.457	32.5 (5.1)	35.1 (4.9)	0.01
LA1 (mm)	44 (8.5)	46.6 (8.0)	0.168	43.1 (8.7)	45.2 (8.8)	0.06
LA2 (mm)	49 (10.2)	52.1 (10.1)	0.166	54.1 (8.7)	53.9 (9.1)	0.09
RA1 (mm)	42.7 (6.3)	42.4 (7.1)	0.451	39.5 (6.7)	43.9 (5.9)	0.01
RA2 (mm)	49.3 (9.1)	47.3 (7.9)	0.254	53.5 (6.9)	55.1 (7.1)	0.144
RASA (mm2)	2131.1 (644.1)	1916.2 (577.6)	0.134	2314.2 (498.2)	2278.5 (514.3)	0.646
Delta EF	2.8 (19.4)	−12.1 (3.2)	0.009			

EF: ejection fraction; TAPSE: tricuspid anular plane systolic excursion; LA: left atrium; LVEDD: left ventricle end-diastolic diameter; LVESD: left ventricle end-systolic diameter; RA: right atrium; RV: right ventricle; RASA: right atrium systolic area.

**Table 3:** Respiratory parameters.

Respirator Parameter	Non-VTI Growth (*n* = 17))	VTI Growth (*n* = 24)	*p*
Mechanical ventilation over than 24 h	7 (41.1%)	13 (54.1%)	0.091
Resp. time (hours)	20.9 (2.1)	25 (3.2)	0.081
Tidal volume (mL)	470.5 (43.5)	490.5 (44.0)	0.079
RR (/min)	13.4 (2.0)	12.9 (1.9)	0.122
PEEP (cmH2O)	6.5 (1.3)	7.7 (1.9)	0.003
FIo2 (%)	38.5 (4.5)	39 (4.8)	0.051

Resp: respiratory; RR: respiratory rate; PEEP: positive end-expiratory pressure; FIo2: fraction of inhaled oxygen; cmH2O: centimeters of water.

**Table 4:** Linear regression of the delta VTI ratio (postop–preop retrograde/anterograde VTI ratio).

Title	B	95% CI		*p* Value
Delta EF	−0.099	−0.022	−0.002	0.022
Fluid balance/body weight at POP 24 h (mL/kg)	0.011	0.001	0.021	0.022
CVP POP 24 h (mmHg)	0.094	0.052	0.213	0.002

EF: ejection fraction; CVP: central venous pressure; POP 24 h: postoperative 24 h.

## Data Availability

The data presented in this study are available on request from the corresponding author. The data are not publicly available due to privacy reasons.

## Supplementary Materials

The following supporting information can be downloaded at: https://www.mdpi.com/article/10.3390/diagnostics12123175/s1. Supplementary files S1–S3 can be found at the website related to this article S1: Consort checklist, S2: Hepatic venous flow, S3: Laboratory parameters.
